# An Aqueous Orange II/γ-Cyclodextrin Complex: Calculation of the Guest/Host Binding Constant and the Superstructure Rod Length

**DOI:** 10.1021/acs.chemmater.6c01136

**Published:** 2026-07-14

**Authors:** David W. Jenkins, Mohan Srinivasarao

**Affiliations:** † DWJ-Analytix LLC, Clayton, North Carolina 27520, United States; ‡ School of Material Science and Engineering, 1372 Georgia Institute of Technology, Atlanta, Georgia 30332, United States; § School of Chemistry and Biochemistry, Georgia Institute of Technology, Atlanta, Georgia 30332, United States

## Abstract

An anisotropic phase can form in water when orange II (O–II) and γ-cyclodextrin (γ-CD) are each present at 0.025 M or higher. Anisotropy is not readily observed in water with only one component present. Both components are needed to observe anisotropy due to a complexation that occurs where 2 orange II molecules are included within the γ-cyclodextrin cavity that subsequently builds into a larger rod-like superstructure. The naphthol moiety has been considered to be the most likely portion of O–II to reside within the cavity, which was further confirmed by birefringence observations with various derivatives of O–II in the presence of aqueous γ-CD. Further understanding of the equilibrium binding constant of the O–II_2_:γ-CD complex was obtained through conductivity titrations in comparison to those in the literature with UV–vis studies. Using binding constants from both approaches, calculations of the O–II_2_:γ-CD concentration were made at the isotropic/anisotropic transition for a variety of solution compositions. Using the O–II_2_:γ-CD concentrations, subsequent calculations were made to predict superstructure rod lengths based on various levels of participation of the O–II_2_:γ-CD complex in rod formation. In comparison to dynamic light scattering measurements, rod lengths on the order of 300 nm may occur where 50–75% of O–II_2_:γ-CD could be involved in rod formation.

## Graphical abstract

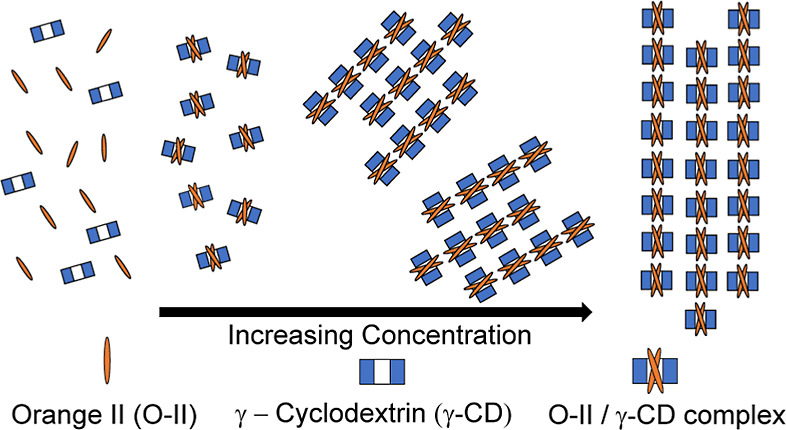

## Introduction

Suzuki et al. reported the formation of a birefringent solution when Orange II and γ-cyclodextrin are dissolved in water where the concentration of each compound is greater than ∼0.03 M.^1,2^ Chemical structures of Orange II (O–II) and γ-cyclodextrin (γ-CD) are provided in Figure 1. Characteristic features of the aqueous O–II/γ-CD mixtures are noticeable viscosity increases and the appearance of a striated texture, easily observable to the naked eye. Striations form once both components are dissolved, indicating an extremely fast self-assembling process for this supramolecular system.

**Figure 1:**
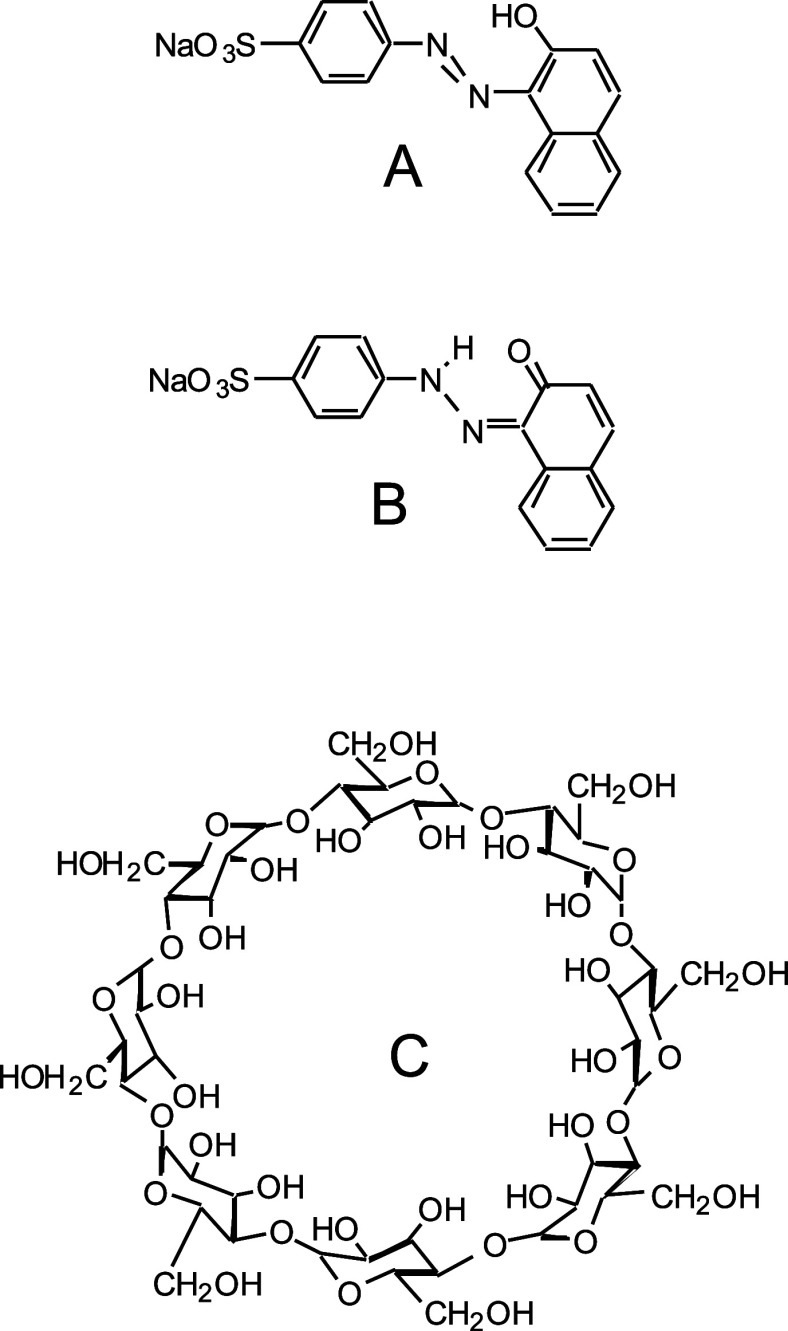
Chemical structures of the azo (A) and hydrazone (B) tautomers of Orange II and γ-cyclodextrin (C).

Other types of self-assembling liquid crystals are known such as chromonic^3–5^ or surfactant-based^6,7^ lyotropic systems, which can occur from a single molecular species that self-assembles into an anisotropic phase. For this O–II/γ-CD system, both components must be present in the solution for the anisotropic phase to form. For example, separate solutions of 0.05 M O–II (aq.) and 0.05 M γ-CD (aq.) are clear (red) and clear (colorless) solutions, respectively, which exhibit no observable striations or birefringence. However, for an aqueous solution comprised of O–II and γ-CD, each at 0.05 M, the viscous, striated solution is readily formed, as described earlier, exhibiting a high level of birefringence.

In earlier work, we further examined how O–II and γ-CD interact with each other in water to form the liquid crystalline-like medium through a 2:1 O–II/γ-CD guest–host complex that further assembles into a superstructure that is considered to be rod-like in nature.^8^ This work utilizes binding constants determined from UV–vis and conductivity studies to calculate the concentration of the 2:1 O–II/γ-CD complex that is then subsequently used to estimate the rod length of the superstructure.

## Experimental Section

### Materials

Orange II (Aldrich 87%) was obtained and purified as previously described.^8^ γ-Cyclodextrin (Wacker-Chemie, Cavamax W8 Food grade, 98% dry weight) was used as received. Purities for O–II and γ-CD were determined to be 94.6 and 91.3%, respectively.^8^

## Methods

Optical micrographs were obtained with a Leica DMRX polarized light microscope and a Sony DKC-5000 Digital Photo Camera. Sample cells were constructed by spacing the coverslip and microscope slide with a 200 μm layer of Teflon tape and sealed with water-resistant epoxy. UV–vis spectra were obtained on a Shimadzu UV-1601 PC spectrophotometer at 25 °C in distilled water using a quartz cell with a 1 cm path length. Conductivity measurements were made in deionized water at 25 °C using an Accumet Research AR50 unit with an Accumet Conductivity Cell (1.0 cm^–1^ cell constant) standardized using a Fisher 1000 μS/cm calibration solution. Calculations of the concentration of the 2:1 O–II/γ-CD complex were conducted using Microsoft Excel Solver by varying the complex concentration to yield the value of the equilibrium binding constant, with the criteria that the concentration of the complex was less than half the initial concentration of O–II.

## Results and Discussion

### Anisotropic Nature and Impact of the Dye Structure

Figure 2 shows an optical micrograph of a sample of 0.05 M (aq.) O–II and 0.05 M (aq.) γ-CD taken between crossed polarizers. The fibrous, banded texture is typically observed for this system, where the bright regions pass through four periods of extinction upon complete rotation of the microscope stage. The naphthol group has been identified as the key O–II moiety considered to reside within the γ-CD cavity.^9,10^ Optical micrographs in Figure 3 further support this because of the birefringence shown in the presence of γ-CD for derivatives of O–II with no additional substituents on the naphthol group, contrary to those derivatives with further functionality on the naphthol.

**Figure 2:**
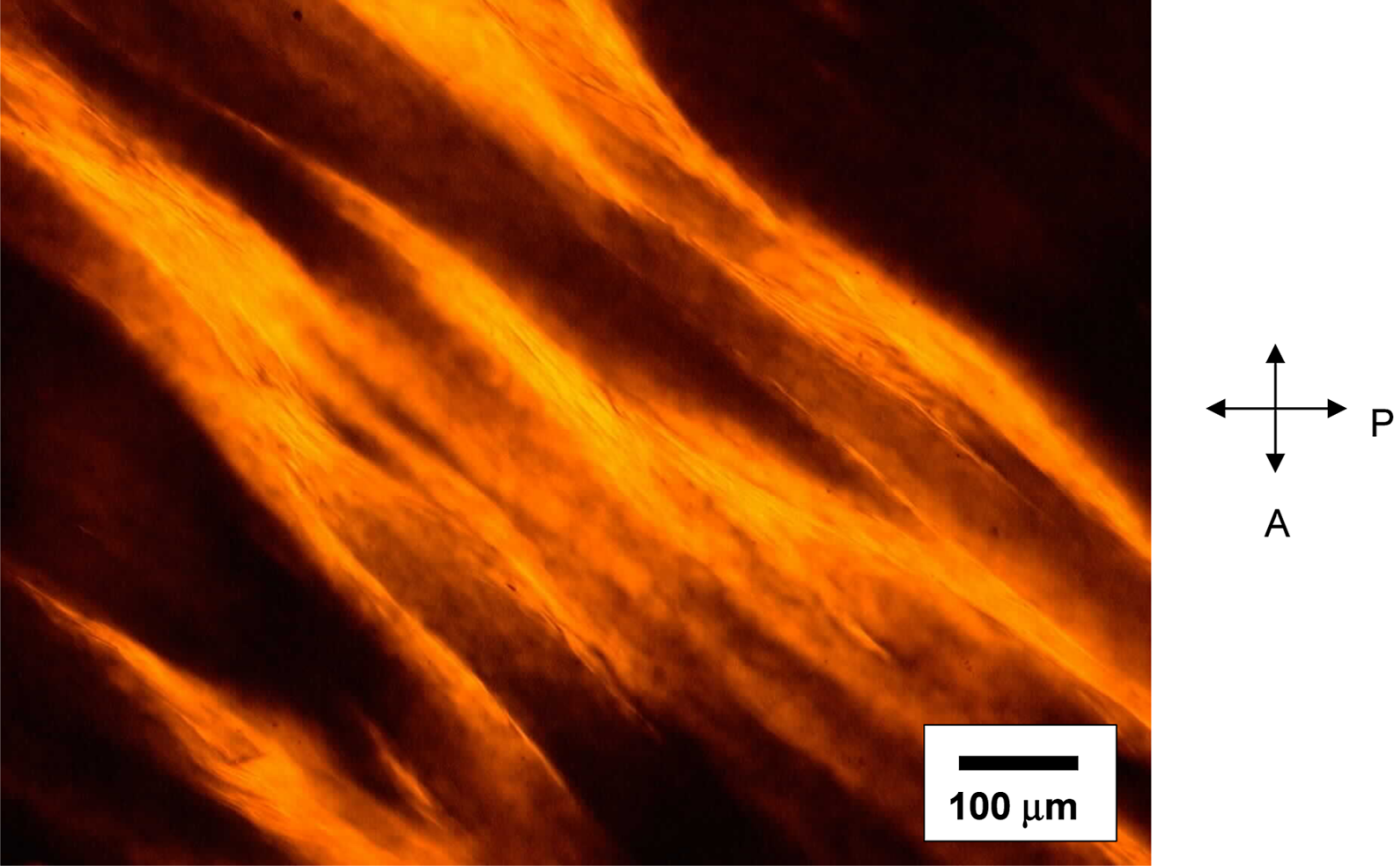
Optical micrograph of 0.05 M (aq.) Orange II with 0.05 M (aq.) γ-cyclodextrin viewed between crossed polarizes.

**Figure 3:**
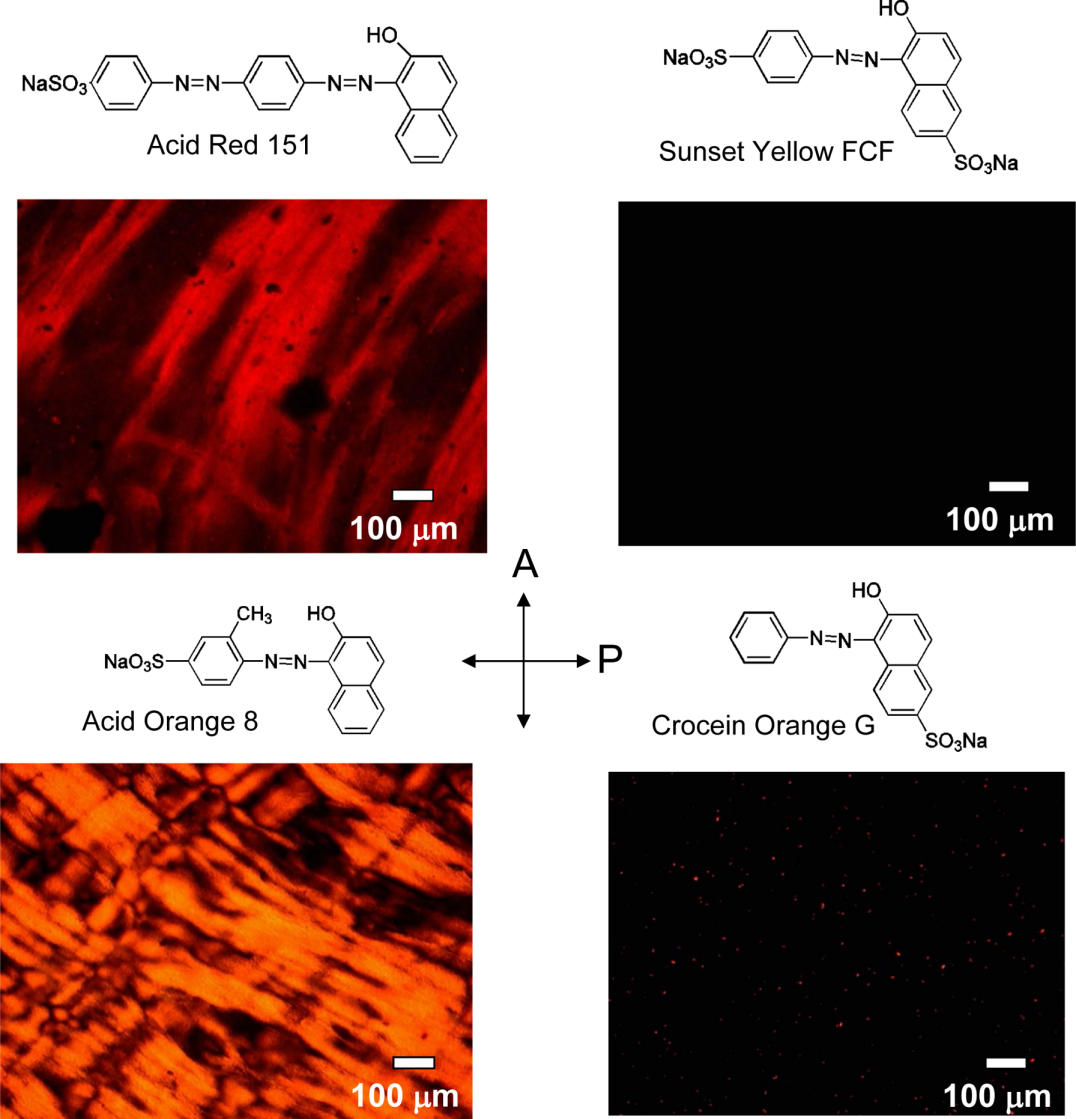
Optical micrographs taken between crossed polarizers of different derivatives of O–II (purified in the same manner as O–II) at 0.05 M (aq.) with 0.05 M (aq.) γ–CD, showing the absence of anisotropy with additional substituents on the naphthalene moiety.

### UV–vis Spectroscopy

Figure 4 shows the UV–vis spectrum of O–II at 5 × 10^–5^ mol/L (aq.) in the presence of increasing amounts of γ-CD. The main effect of the addition of γ-CD is the decrease in the molar absorptivity of O–II in the range of 440–520 nm. Clarke et al. observed the same effect with this system and attributed the observation predominantly to the inclusion of an O–II dimer within the γ-CD cavity, primarily a 2:1 complex; it was concluded that 1:1 and 2:2 complexes were possible as well, but to a much lesser degree.^11^ Due to their amphiphilic nature, azo dyes such as O–II are believed to aggregate with increases in concentration.^12^ Reeves et al. observed that increasing the concentration of O–II (aq.) from 4 × 10^–6^ M to 1 × 10^–3^ M caused the molar absorptivity of the band from approximately 440–520 nm to decrease significantly (centered at 480 nm, from 2.3 × 10^4^ to 1.9 × 10^4^ M^–1^ cm^–1^) in the same manner as observed in Figure 4, which was attributed to the formation of dimers and possibly higher-order aggregates of O–II.^13^ Based on a ^1^H NMR study, a proposed geometry of the O–II dimer was an antiparallel stack to relieve Coulombic repulsions from the sulfonate groups.^14^ Coincidentally, the proposed structure of the 2:1 O–II/γ-CD complex was that of an O–II dimer in an antiparallel stack within the γ-CD cavity;^11^ however, additional geometries of the 2:1 O–II/γ-CD complex were discussed with the naphthol group with the CD cavity^8^ based on the calculated angle by Reeves et al. between the O–II molecules within the dimer.^13^

**Figure 4:**
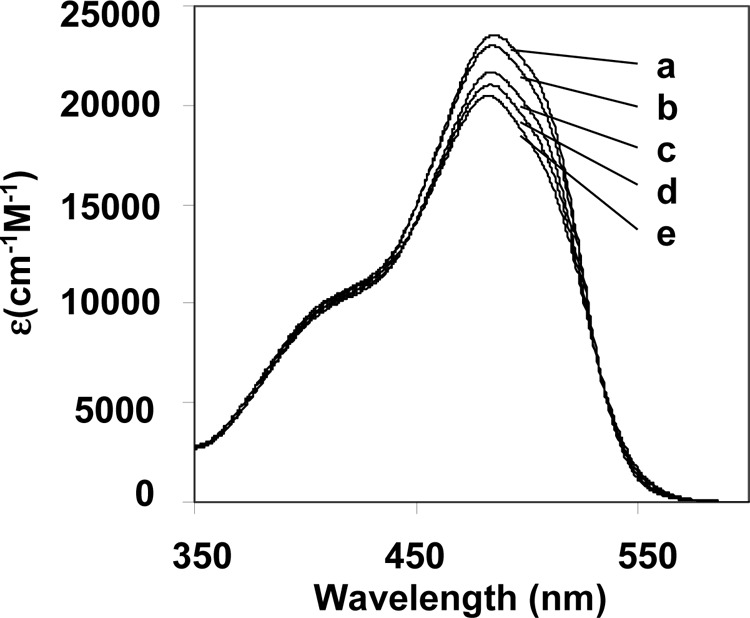
UV–vis spectra of Orange II with increasing amounts of γ-cyclodextrin. [O–II] = 5 × 10^–5^ mol/L, constant for all spectra; [γ-CD] mol/L = (a) 0, (b) 1.6 × 10^–5^, (c) 2.5 × 10^–5^, (d) 5.0 × 10^–5^, and (e) 1.0 × 10^–4^.

Regardless of the exact geometry of the 2:1 O–II/γ-CD complex, Clarke et al. conducted temperature-jump studies on this system and suggested that the following series of complexation occur with the corresponding equilibrium constants.^11^

1 $$O-II+\gamma -CD⇌O-II:\gamma -CD\;K_{1}=4.18\pm 1.47x10^{2}L/mol$$

2 $$O-II+O-II:\gamma -CD⇌{O-II}_{2}:\gamma -CD\;K_{2}=1.68\pm 0.54x10^{6}L/mol$$

3 $$O‐II_{2}:\gamma ‐CD+\gamma ‐CD⇌O‐II_{2}:\gamma ‐CD_{2}\;K_{3}=1.77\pm 1.54x10^{2}L/mol$$

Based on these equilibrium constants, it was calculated that while operating at a [γ-CD]_o_/[O–II]_o_ (o subscript denotes total concentration) less than 10, OII_2_: γ-CD is clearly the dominant complex.

#### Fluorescence Spectroscopy

Orange II is known to exist in an equilibrium between its respective azo (Figure 1a) and hydrazone (Figure 1b) tautomeric states. For O–II, absorptions in the range of 350–440 nm and 440–550 nm are attributed to the azo and hydrazone tautomers, respectively.^15–19^ Figure 4 shows a predominance of the hydrazone tautomer based on this band assignment, which is consistent with the determination of Lycka et al. for 1-phenylazo-2-naphthol primarily existing (∼70+%) as the hydrazone tautomer.^20–22^ Ibanez et al. studied the fluorescence of O–II in water and suggested that O–II can undergo an excited-state intramolecular proton transfer (ESIPT).^23^ A characteristic of O–II that facilitates ESIPT is the presence of a functional group involved in tautomerism that is capable of forming an intramolecular hydrogen bond.^24^ Ibanez et al. suggest that the hydrazone tautomer of O–II is the fluorescent species and that excitation with light absorbed by the azo tautomer induces the ESIPT to the hydrazone, which subsequently emits. It was observed that O–II (5.3 × 10^–5^ M) emits with a λ_max_ of 560 nm whether excited with 400 nm (azo absorption) or 480 nm (hydrazone absorption).^23^ Although the previous observation may not be direct evidence for ESIPT since emission can be independent of excitation wavelength, it should be noted that the methoxy derivative of 1-phenylazo-2-naphthol (which cannot form the hydrazone tautomer) does not fluoresce.^25^

The effect of O–II and γ-CD concentration on the fluorescence of O–II was studied with 484 nm excitation in earlier work.^8^ Two major observations were made regarding the position of λ_max_. Having O–II at a more relatively dilute concentration, the addition of γ-CD red shifts the emission spectrum from 560 nm up to 600 nm. Even in the absence of γ-CD, increasing the concentration of O–II red-shifts the emission spectrum to 600 nm. Both observations could be attributed to excimer fluorescence from the O–II dimer. The observation that increases in γ-CD concentration promote the same spectral changes in the emission of O–II at relatively dilute solutions as increasing O–II concentration does complements the UV–vis results previously discussed and thus provides further evidence that an O–II_2_:γ-CD complex is formed under these conditions.

### Conductometric Titration

Conductometric titrations have been used as a means of determining the stoichiometry and binding constants for the inclusion of compounds with ionic functionality, namely, aromatic salts^26–29^ and surfactants,^30,31^ within various cyclodextrins. The premise behind this method is that the conductivity of a solution containing a constant amount of the ionic guest will decrease with the addition of cyclodextrin as it is included within the cavity. The position of any inflection point observed in the titration curve can be used to determine the stoichiometry of the complex, while the sharpness of the inflection point is related to the strength of the binding constant; this point is well demonstrated in work by Junquera et al.^29^

Figure 5 plots the original and temperature-corrected (25 °C) specific conductivity (κ, mS/cm) of an aqueous O–II solution ([O–II]_o_ = 0.02 M) as a function of added γ-CD (dry powder), where the abscissa is actually shown as the mole ratio of γ-CD/O–II. As shown in earlier work by Srinivasarao and Jenkins, an inflection point is observed at a mole ratio of γ-CD/O–II of approximately 0.5, offering further evidence for the formation of a 2:1 O–II/γ-CD complex.^8^ In addition, Junquera et al. studied the complexation of amitriptyline (AMYTP), a tricyclic antidepressant, with β-CD using fluorescence spectroscopy and conductometric titrations.^29^ β-CD has the same structure as γ-CD (Figure 1c) except that it is comprised of seven glucose repeat groups rather than eight. The conductometric titration of an aqueous solution of AMYTP with β-CD showed an inflection point similar to that of Figure 5 except that the position of the inflection point was at a mole ratio of β-CD/AMYTP of 1. Furthermore, the fluorescence spectrum of AMYTP exhibited no shifting of λ_max_ upon the addition of β-CD.

**Figure 5:**
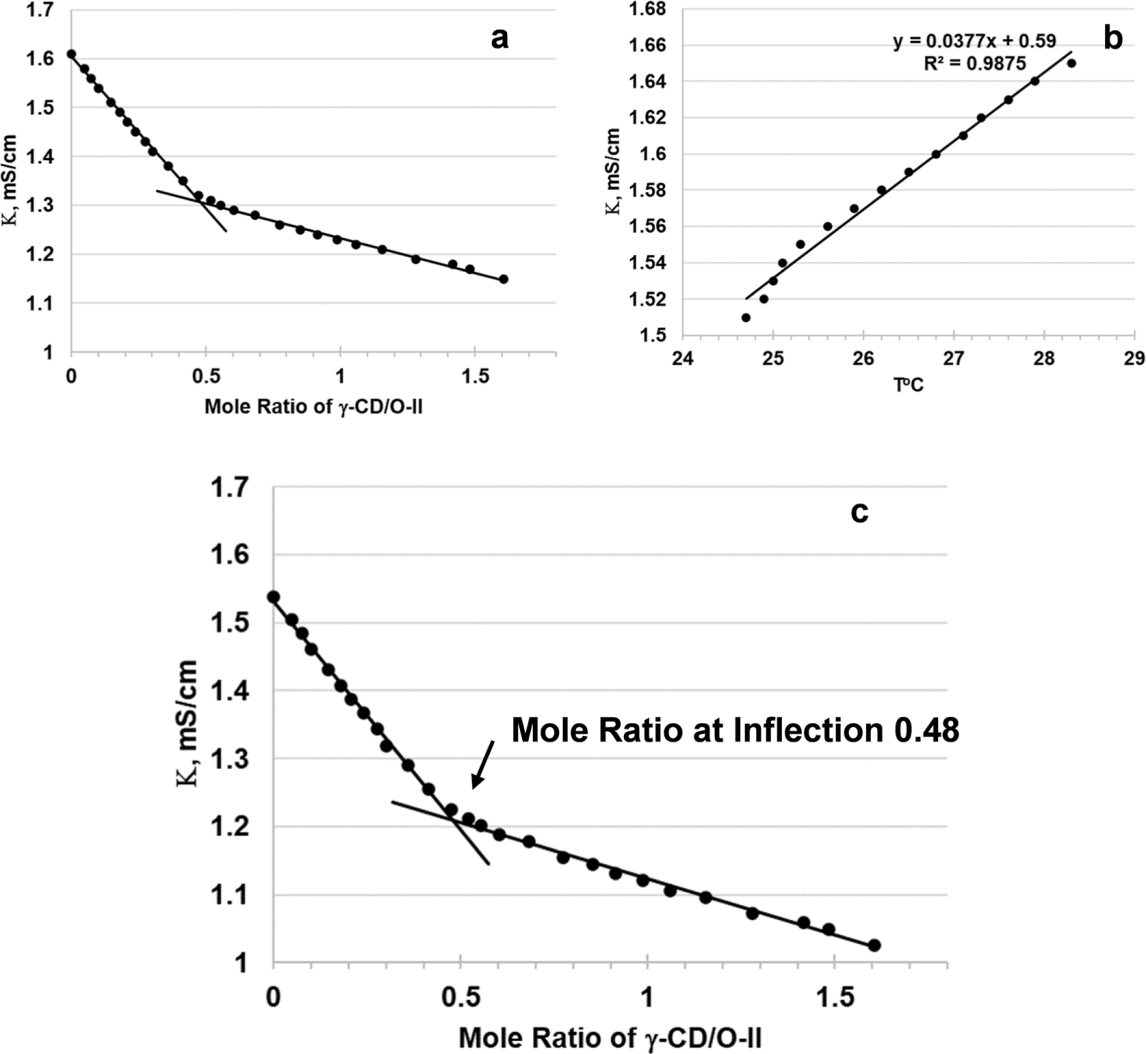
Specific conductivity (κ) of 0.02 M O–II (aq.) as a function of added γ-CD powder. From solution agitation, the temperature began above 25 °C and increased slightly with powder addition in the original data (a). A separate solution of O–II at 0.02 M O–II was monitored at different temperatures (b) to approximate the basic change of conductivity as a function of temperature to generate a temperature-corrected curve (c) to 25 °C.

## Binding Constant Determination from Conductivity Measurements

Considering the methodology outlined in previous works,^26–29^ the determination of the binding constant (K_eq_) from conductometric titrations for the complexation of O–II with γ-CD leading to a 2:1 O–II/γ-CD complex is outlined below. Considering the proposed equilibrium in eq 4, the specific conductivity (κ, S/cm) of an

4 $$2O-II+\gamma -CD⇌{O-II}_{2}:\gamma -CD$$

aqueous solution of O–II in the presence of γ-CD is a summation of the contributions from each of the conductive species as shown in eq 5, where molar conductivities, λ_*x*_, and

5 $$\kappa =\left[{Na}^{+}\right]\lambda_{Na+}+\left[{O-II}^{-}\right]\lambda_{O-II-}+\left[{O-II}_{2}^{-2}:\gamma -CD\right]\lambda_{{O-II}_{2}^{-2}:\gamma -CD}$$

concentrations, [X], for each species, X, are in units of S cm^2^/mol and mol/cm^3^, respectively. Equation 5 assumes that contributions from other possible types of O–II/γ-CD complexes, namely, 1:1 and 2:2, are negligible. Furthermore, the species noted as O–II^–^ is assumed to be either monomeric or aggregated O–II that is not complexed with γ-CD. The total concentrations of O–II and γ-CD can be described by eqs 6 and 7.

6 $${\left[O-II\right]}_{o}=\left[{Na}^{+}\right]=\left[{O-II}^{-}\right]+2\left[{O-II}_{2}^{-2}:\gamma -CD\right]$$

7 $${\left[\gamma -CD\right]}_{o}=\left[\gamma -CD\right]+\left[{O-II}_{2}^{-2}:\gamma -CD\right]$$

The value of K_eq_ will be determined by the concentrations of each component in eq 4 and their respective activity coefficients, f_n_, as shown in eq 8.

8 $$K_{eq}=\frac{\left[{O-II}_{2}^{-2}:\gamma -CD\right]f_{{O-II}_{2}^{-2}:\gamma -CD}}{{\left[{O-II}^{-}\right]}^{2}f_{{O-II}^{-}}^{2}\left[\gamma -CD\right]f_{\gamma -CD}}$$

Activity coefficients for each component at 25 °C can be estimated using eq 9, where z is the valence of the different components and I is the ionic strength.^32^

9 $$\log\left(f_{n}\right)=\frac{-0.5115\left|z_{1}z_{2}\right|\sqrt{I}}{1+\sqrt{I}}+0.1\;\left|z_{1}z_{2}\right|\;I$$

Definitions and values for variables in eq 9 used in this work, including calculated activity coefficients, are provided in Table 1 (f_γ‑CD_ is assumed to be 1.0 due to its lack of charge).

**Table 1:** Values and Definitions of Variables Used for the Calculation of the Binding Constant for the O–II/γ-CD Inclusion Complex

variable	definition	value
z_Na+_	valence	+1
z_O–II_^–^	valence	–1
z_O–II2_^–2^_: γ‑CD_	valence	–2
I_O–II_	ionic strength for 0.02 M O–II	0.02 M
I_O–II2: γ‑CD_	ionic strength for 0.01 M O–II_2_^–2^:γ-CD (assuming full complexation from 0.02 M O–II)	0.03 M
f_O–II‑_	Act. Coeff. of O–II^–^	0.868 (calculated from eq 9)
f_O–II2_^–2^_: γ‑CD_	Act. Coeff. of O–II_2_^–2^:γ-CD	0.716 (calculated from eq 9)
λ_Να+_^ο^	molar conductivity of Na^+^ at infinite dilution	50.1 S cm^2^/mol^28^
a_Na+_	estimated size of Na^+^	4 Å^28^
C	concentration of Na^+^	0.02 M
D	rod diameter in nm assumed from the diameter of γ-CD	1.75 ^35^
l	length of O–II_2_:γ-CD in nm within a rod assumed to be approximately the same as the diameter of γCD + Na^+^	2.15

Using eq 10,^32^ λ_Na+_ has been calculated to be 41.5 S cm^2^/mol at 25 °C, where a and c represent the atomic size and concentration, respectively, for Na^+^ with the values for parameters used provided in Table 1.

10 $$\lambda_{Na+}=\lambda_{Na+}^{o}-\frac{\left(60.65+0.23\lambda_{Na+}^{o}\right)\sqrt{c}}{\left(1+0.3291a_{Na+}\sqrt{c}\right)}$$

Since the specific conductivity is only attributed to Na^+^ and O–II^–^ before any γ-CD is added to the solution, λ_O–II‑_ has been calculated to be 35.4 S cm^2^/mol from the first data point of Figure 5 and the calculated value for λ_Na+_.

Considering eqs 5–8, the remaining parameters to be determined are [O–II^–^], [γ-CD], [O–II_2_^–2^: γ-CD], K_eq_, and λ_O–II2–2: γ‑CD_. Knowledge of λ_O–II2–2: γ‑CD_ will allow these unknowns to be solved for each data point in the titration curve of Figure 5. Unfortunately, the value of λ_O–II2–2: γ‑CD_ cannot be directly determined from the data but rather has to be estimated from extrapolation of the latter portion of the titration curve in Figure 5. Before conducting the calculations to determine K_eq_, a modification to the raw data in Figure 5 needs consideration.

The premise behind this technique is that the primary decrease in conductivity is due to the inclusion of O–II within the γ-CD cavity, resulting in a decrease in the diffusivity of the conductive species. However, the addition of cyclodextrin to an aqueous solution of inorganic electrolyte can cause a reduction in conductivity even though the electrolyte is not believed to bind or be included within the cavity. This effect, attributed to a viscosity increase upon the addition of cyclodextrin, was acknowledged in binding studies of ionic biphenyl compounds with α-CD.^26^ The decrease in conductivity of aqueous NaCl solutions when titrated with α-CD was quantified and used as a correction for determining the binding constant.^26^ Figure 6 demonstrates this effect on aqueous NaCl solutions titrated with powdered glucose, α-CD, and γ-CD. The glucose titration is actually plotted as equivalents of α-CD and γ-CD to demonstrate that the addition of glucose can cause a similar decrease in conductivity as does cyclodextrin. Besides viscosity changes as suggested by Gelb et al.,^26^ the addition of carbohydrate could decrease the solution conductivity as a result of dilution and from potentially decreasing the dielectric constant of the medium.

**Figure 6:**
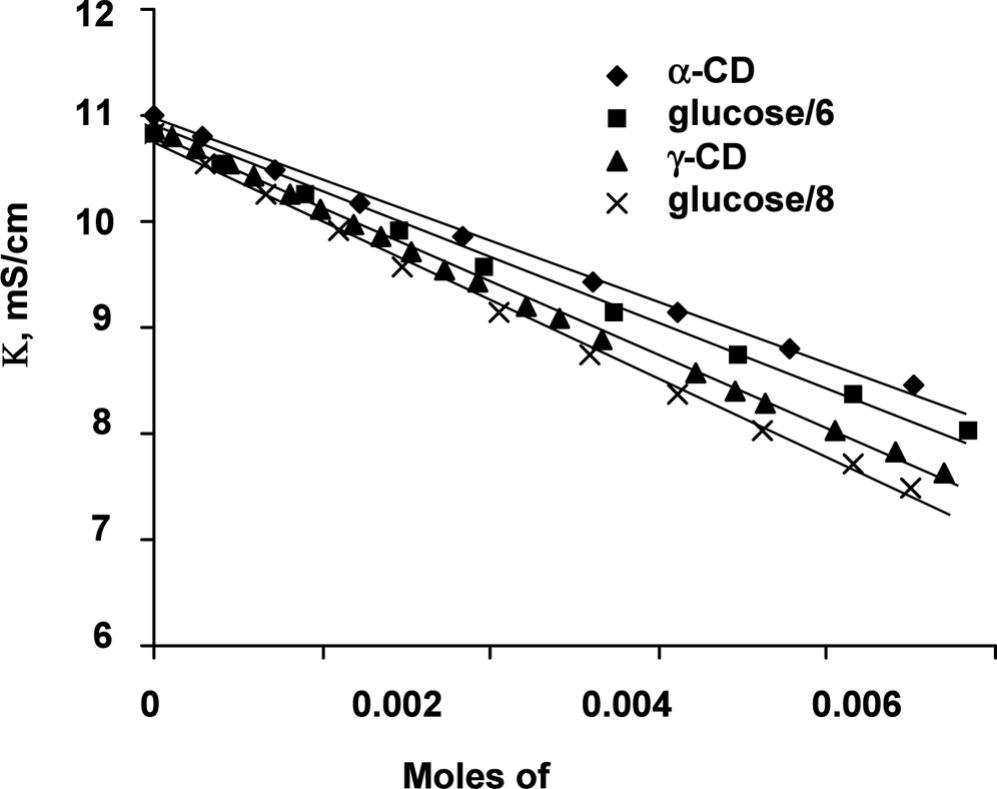
Specific conductivity (κ) of 0.1 M NaCl (aq.) at 25 °C as a function of added α-CD, γ-CD, and glucose powder.

Thus, an aqueous solution of 0.02 M O–II was titrated with glucose and plotted as equivalents of γ-CD as shown in Figure 7. Using the slope from the glucose titration, the data in Figure 5 was corrected to that shown in Figure 7 using eq 11, where κ_c_, κ_o_, and MR are the

11 $$\kappa_{c}=\kappa_{o}+0.1132\left(MR\right)$$

corrected conductivity, original conductivity, and mole ratio of γ-CD/O–II, respectively. Using the last point in the titration for the corrected data, λ_O–II2–2: γ‑CD_ was estimated to be 37.7 S cm^2^/mol in the same manner as used to calculate λ_O–II‑_. Since the complexation is an equilibrium process, the value obtained for λ_O–II2–2: γ‑CD_ is overestimated due to the simultaneous presence of uncomplexed O–II^–^. This value, however, was used as an initial approximation for λ_O–II2–2: γ‑CD_ in order to conduct the calculations. With this initial value for λ_O–II2–2: γ‑CD_, a K_eq_ was calculated for each point in the titration curve, except for the first and last points. Using eqs 5–8, an iterative calculation was conducted where the value of λ_O–II2–2: γ‑CD_ was decreased (to 29.8 S cm^2^/mol) until the K_eq_ calculated for the last point in the curve reached the average value of K_eq_ calculated from all of the points in the titration curve. With this approach, the binding constant for eq 4 has been calculated to be 2.123 × 10^4^ L^2^ mol^–2^ ± 35%.

**Figure 7:**
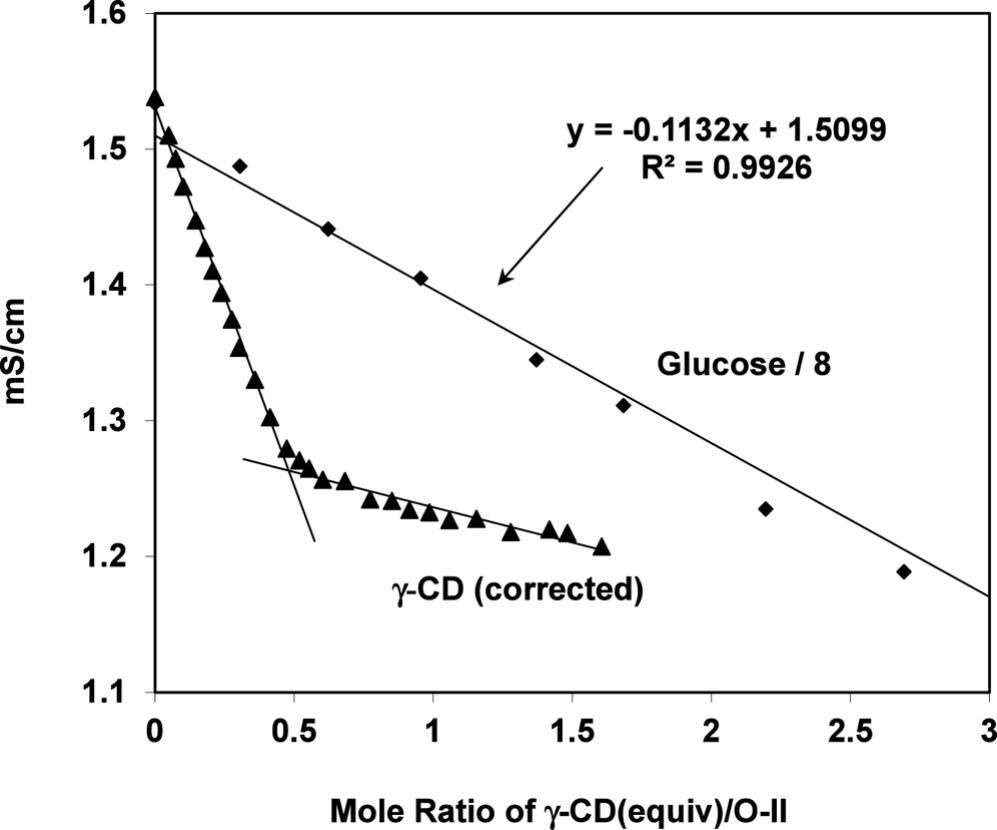
Specific conductivity (κ) of 0.02 M O–II (aq.) at 25 °C as a function of added glucose and γ-CD powder (corrected curve from Figure 5c).

### Determination of Superstructure Rod Length

The determination of the rod length for the superstructure begins with the relationships that describe the volume fraction of rods (Φ) in the isotropic (eq 12) and anisotropic (eq 13) phases of the rod-like nematic system, where D and L are the diameter and length of the rod, respectively.^33,34^

12 $$\Phi_{i}^{c}=\frac{3.3D}{L}$$

13 $$\Phi_{nema}^{c}=\frac{4.5D}{L}$$

The volume fraction of rods can also be described with eq 14, where c is the concentration of rods, considered as the number of rods per volume (N_rods_/V).

14 $$\Phi =\frac{1}{4}\pi LD^{2}c$$

For the 2:1 O–II/γ-CD system, several geometries for the assembly of the rod-like structure have been proposed,^8^ all of which could be envisioned as single 2:1 O–II/γ-CD complexes with length l and diameter D that connect linearly lengthwise to make the larger structure of length L and diameter D. With eq 15, the total number of rods (N_rods_) within the 2:1 O–II/γ-CD system could be estimated, where N_O–II2:γ‑CD_ are the total number of 2:1 O–II/γ-CD

15 $$N_{rods}=\frac{l\left(N_{{O-II}_{2}:\gamma -CD}\right)}{L}$$

complexes that participate in forming rods in the system.

To determine the rod length of superstructures that transition from an isotropic to an anisotropic phase, the concentration of components at the transition was considered. Figure 8 provides additional notations from previous work^8^ to indicate the approximate concentrations (C1 – C8) across the isotropic/anisotropic transition for various solutions at different concentrations and ratios of O–II and γ-CD. From the concentrations estimated at this transition (specifically the initial concentrations, [O–II]_o_ and [γ-CD]_o_), the resulting concentrations of O–II, γ-CD, and 2:1 O–II/γ-CD complexes at equilibrium were calculated as shown in Figure 9 using eqs 6, 7, and 8 and the binding constants determined from conductivity measurements (2.123 × 10^4^ L^2^ mol^–2^) and from Clarke et al.^11^ (7.02 × 10^8^ L^2^ mol^–2^ from eqs 1 and 2 with K_1_ x K_2_). Considering both binding constants, the concentration of the 2:1 O–II/γ-CD complex is higher at ∼ 0.02 M (0.017 M – 0.023M) for the lower ratios of γ-CD/O–II (C1, C2) but lowers to ∼ 0.01 M (0.007 M – 0.012M) for higher ratios (C3–C8). When O–II is present in higher proportions as in C1 and C2, the formation of the 2:1 O–II/γ-CD complex is more prominent because the stoichiometry of the complex drives a higher dependency or influence of O–II.

**Figure 8:**
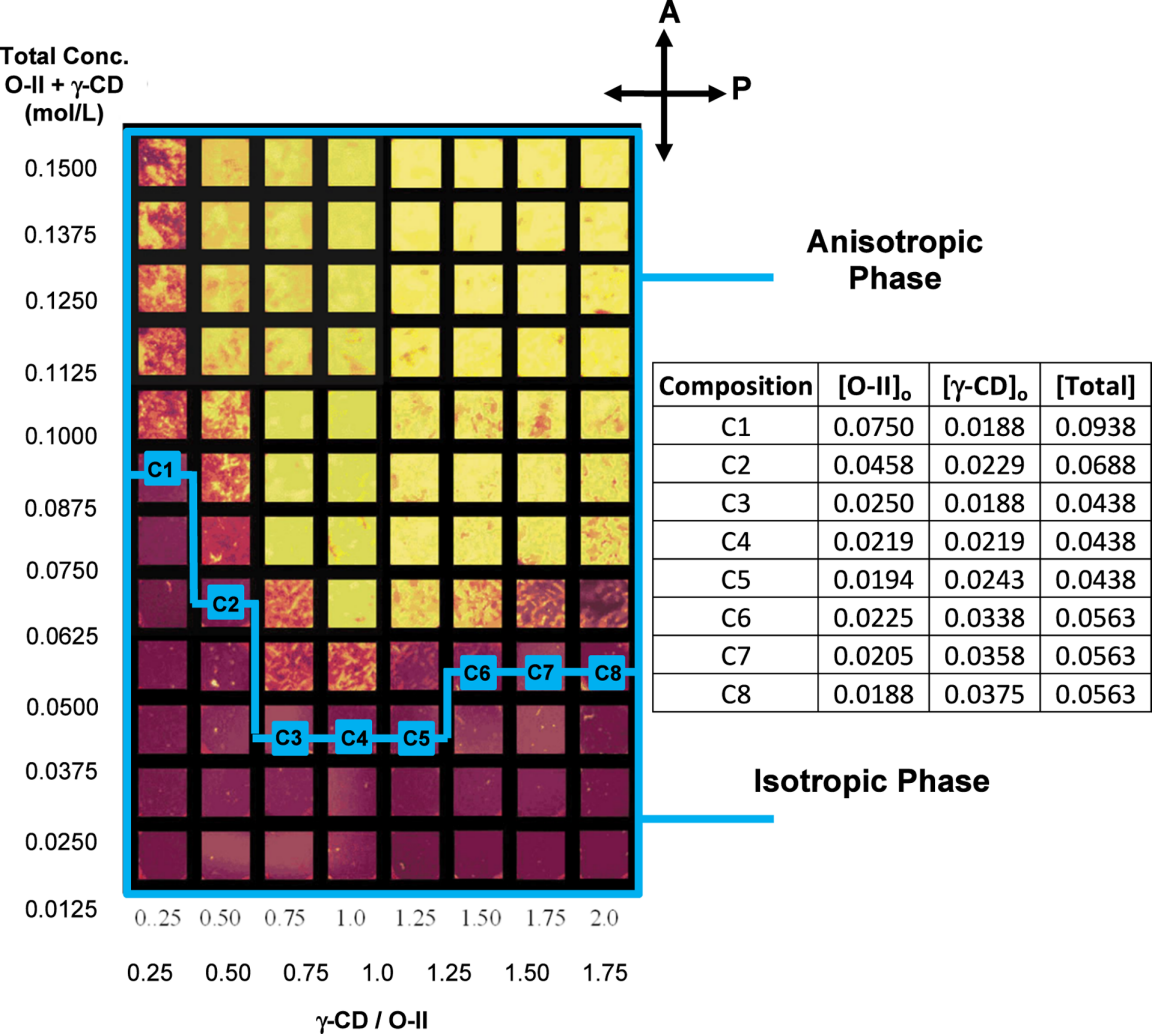
Unmagnified image of various solutions in a 96-well plate at different ratios and concentrations of O–II and γ-CD that were illuminated between crossed polarizers,^8^ with notations for the overall isotropic and anisotropic phases. In the isotropic region, there are a few small birefringent particulates presumed to be imperfections from dust or other artifacts. Judgement of the isotropic/anisotropic boundary (C1 – C8) was based on the general appearance of each well.

**Figure 9:**
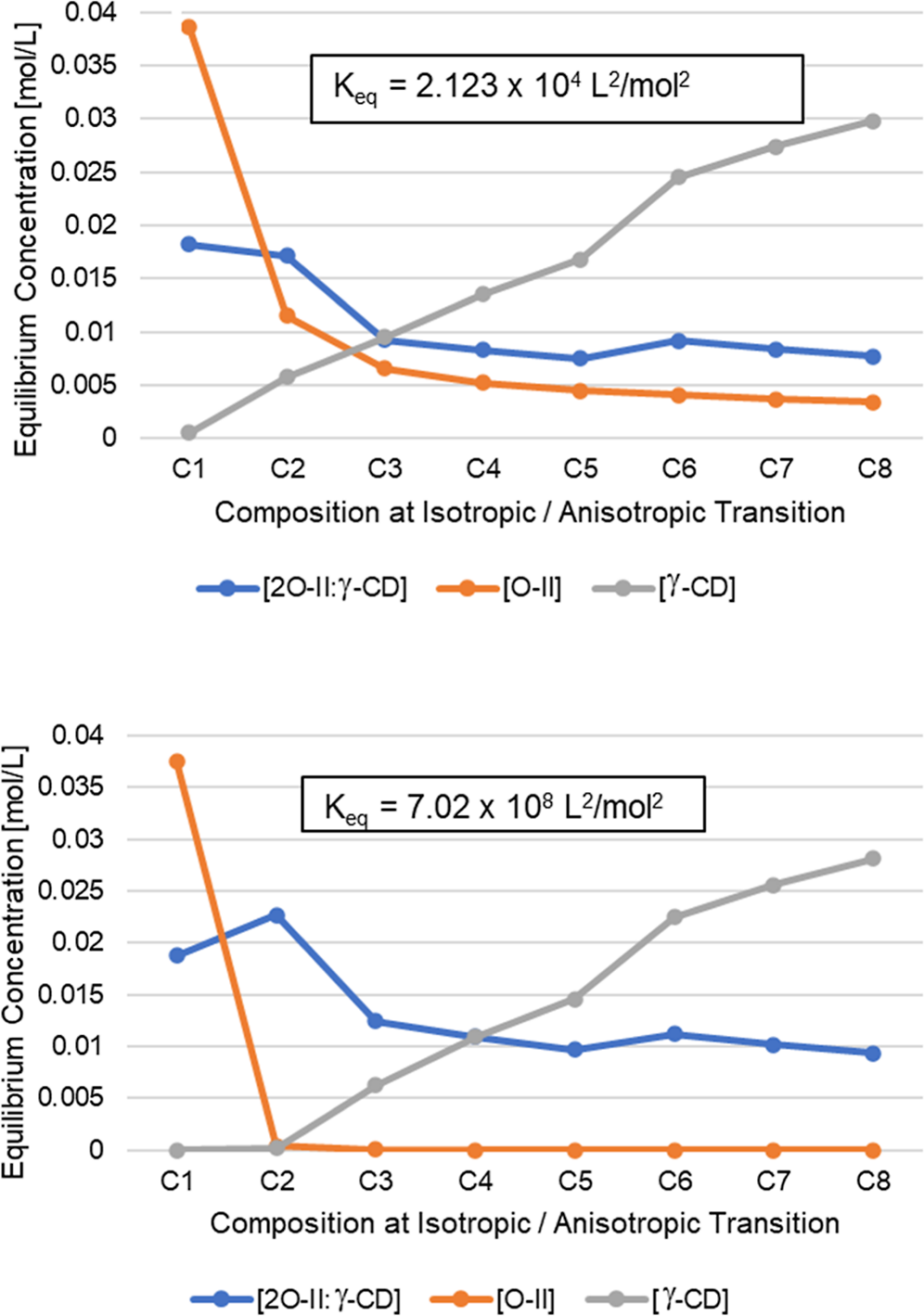
Calculation of O–II, γ-CD, and O–II_2_:γ-CD concentrations as a function of the equilibrium binding constant.

To calculate the effective rod length (L) at the transition from the isotropic phase into the anisotropic, eq 16 is derived by equating eqs 12 to 14 while substituting eq 15 for N_rods_.

16 $$L=3.3D\frac{V}{l\left(N_{{O-II}_{2}:\gamma -CD}\right)}\frac{4}{\pi D^{2}}$$

From the 2:1 O–II/γ-CD complex concentrations calculated for C1–C8 from both binding constants, effective rod lengths were calculated by converting the molar concentration of the 2:1 O–II/γ-CD complex into N_O–II2:γ‑CD_ for a V of 1 L. The calculation shown in eq 16 assumes all O–II_2_:γ-CD complexes participate in rod formation. The proportion of O–II_2_:γ-CD complexes that participate in the assemblage of a rod-like superstructure is unknown and most likely will be dependent on the balance of ionic association/dissociation^36–38^ of the sodium sulfonate group as has been proposed contributes to the rod formation.^8^ With this uncertainty, Figure 10 provides the calculated rod lengths for different percentages of N_O–II2:γ‑CD_ involved in superstructure formation. Generally, rod lengths are projected to be smaller with higher concentrations of the O–II_2_:γ-CD complex and with a higher percentage of participation in rod formation, allowing a balancing of the total volume fraction of rods in solution.

**Figure 10:**
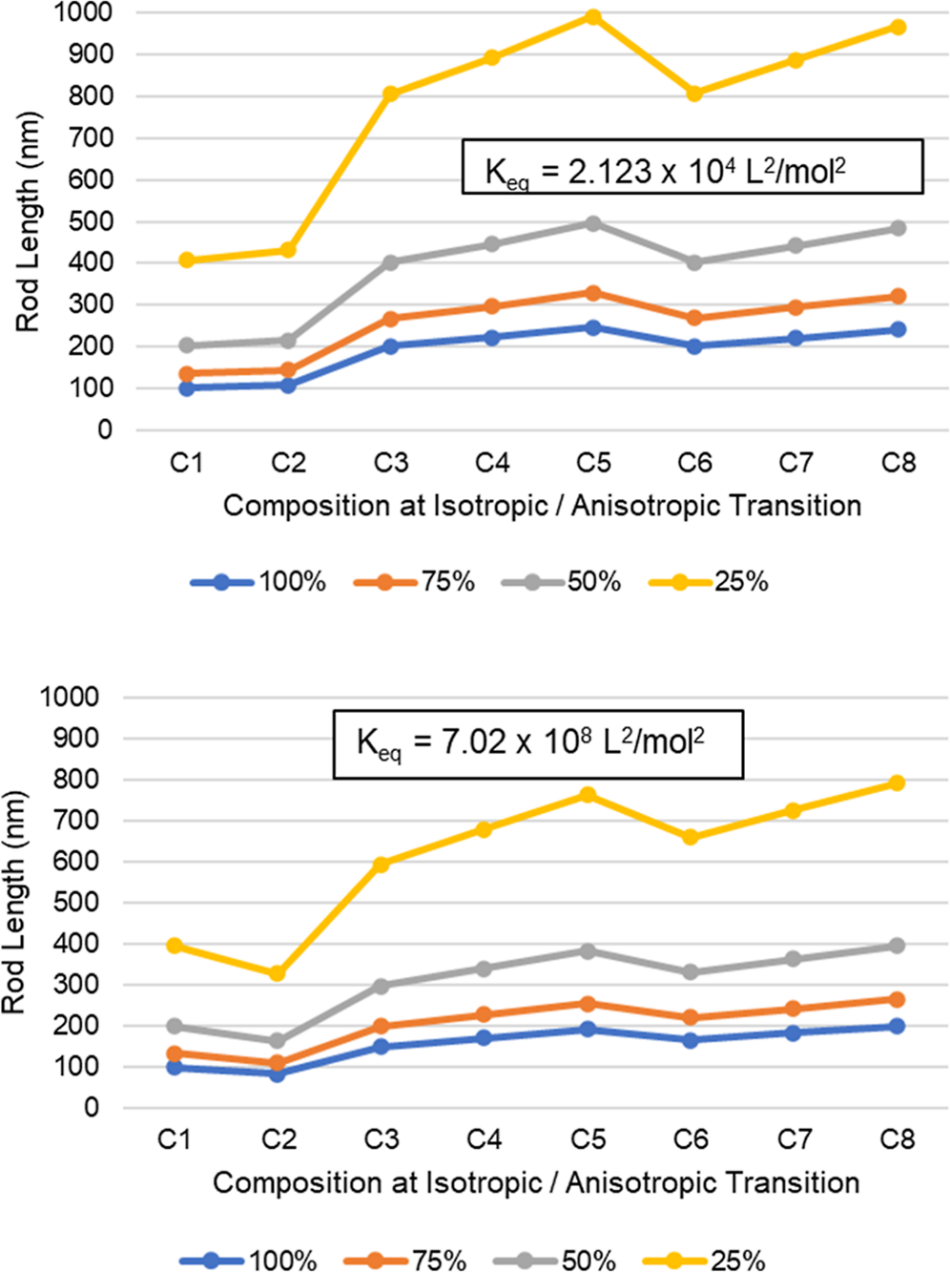
Calculation of effective superstructure rod length as a function of the equilibrium binding constant and percentage of O–II_2_:γ-CD participation in rod formation.

Preliminary dynamic light scattering (unpublished results) of isotropic solutions for this system containing both O–II and γ-CD at 0.015 M and at 0.04 M indicated hydrodynamic radius (*R*_h_) measurements of ∼ 50 nm and ∼ 60 nm, respectively. The *R*_h_ can be related to the effective length (a) and width (b) of a prolate ellipsoid^39^ through eqs 17 and 18.

17 $$R_{h}=\frac{a}{f\left(\frac{b}{a}\right)}$$

18 $$f\left(\frac{b}{a}\right)=\frac{\ln\left(\frac{1+\sqrt{1-\frac{b^{2}}{a^{2}}}}{\left(\frac{b}{a}\right)}\right)}{\sqrt{1-\frac{b^{2}}{a^{2}}}}$$

When the width (b) is equated to 1.75 nm based on the diameter of γ-CD, then *R*_h_ values of 50 and 60 nm predict lengths of 287 and 361 nm, respectively. From Figure 10, these values suggest approximately 50 – 75% involvement of the O–II_2_:γ-CD complex participating in rod formation, which correlates well with degrees of counterion dissociation ranging from ∼ 0.2 to 0.4 for sodium alkyl sulfate systems.^36^

## Conclusions

The likelihood of the naphthol group in O–II residing within the γ-CD to form the O–II_2_:γ-CD was further substantiated by birefringence observations with derivatives of O–II in the presence of γ-CD. An equilibrium binding constant for the O–II_2_:γ-CD complex was determined from conductivity titrations. In comparison to a binding constant available from other UV–vis studies, the concentration of the O–II_2_:γ-CD complex was determined from the binding constant at the isotropic/anisotropic transition point for a variety of solution compositions. From the concentrations determined for the O–II_2_:γ-CD complex, calculations of the effective rod lengths were made for a range of percentages of participation in rod formation of the O–II_2_:γ-CD complex. From dynamic light scattering results, superstructure rod lengths may exist on the order of 300 nm, where 50–75% of the O–II_2_:γ-CD complex contributes to rod formation.
